# Death of Prof. Shotwell

**Published:** 1850-08

**Authors:** 


*Death of Prof. Shotwell.*—We are pained to notice the decease of Prof. John T. Shotwell, of the Medical College of Ohio. He fell a victim to the epidemic cholera.

